# Clarify Sit-to-Stand Muscle Synergy and Tension Changes in Subacute Stroke Rehabilitation by Musculoskeletal Modeling

**DOI:** 10.3389/fnsys.2022.785143

**Published:** 2022-03-14

**Authors:** Ruoxi Wang, Qi An, Ningjia Yang, Hiroki Kogami, Kazunori Yoshida, Hiroshi Yamakawa, Hiroyuki Hamada, Shingo Shimoda, Hiroshi R. Yamasaki, Moeka Yokoyama, Fady Alnajjar, Noriaki Hattori, Kouji Takahashi, Takanori Fujii, Hironori Otomune, Ichiro Miyai, Atsushi Yamashita, Hajime Asama

**Affiliations:** ^1^Department of Precision Engineering, The University of Tokyo, Tokyo, Japan; ^2^Department of Information Science and Electrical Engineering, Kyushu University, Fukuoka, Japan; ^3^RIKEN Center for Brain Science, Aichi, Japan; ^4^Department of Physical Therapy, Saitama Prefectural University, Saitama, Japan; ^5^College of Information Technology, United Arab Emirates University, Al Ain, United Arab Emirates; ^6^Department of Rehabilitation, University of Toyama, Toyama, Japan; ^7^Morinomiya Hospital, Osaka, Japan

**Keywords:** muscle tension, muscle synergy, sit-to-stand (STS), stroke, subacute rehabilitation, EMG normalization, musculoskeletal modeling

## Abstract

Post-stroke patients exhibit distinct muscle activation electromyography (EMG) features in sit-to-stand (STS) due to motor deficiency. Muscle activation amplitude, related to muscle tension and muscle synergy activation levels, is one of the defining EMG features that reflects post-stroke motor functioning and motor impairment. Although some qualitative findings are available, it is not clear if and how muscle activation amplitude-related biomechanical attributes may quantitatively reflect during subacute stroke rehabilitation. To better enable a longitudinal investigation into a patient's muscle activation changes during rehabilitation or an inter-subject comparison, EMG normalization is usually applied. However, current normalization methods using maximum voluntary contraction (MVC) or within-task peak/mean EMG may not be feasible when MVC cannot be obtained from stroke survivors due to motor paralysis and the subject of comparison is EMG amplitude. Here, focusing on the paretic side, we first propose a novel, joint torque-based normalization method that incorporates musculoskeletal modeling, forward dynamics simulation, and mathematical optimization. Next, upon method validation, we apply it to quantify changes in muscle tension and muscle synergy activation levels in STS motor control units for patients in subacute stroke rehabilitation. The novel method was validated against MVC-normalized EMG data from eight healthy participants, and it retained muscle activation amplitude differences for inter- and intra-subject comparisons. The proposed joint torque-based method was also compared with the common static optimization based on squared muscle activation and showed higher simulation accuracy overall. Serial STS measurements were conducted with four post-stroke patients during their subacute rehabilitation stay (137 ± 22 days) in the hospital. Quantitative results of patients suggest that maximum muscle tension and activation level of muscle synergy temporal patterns may reflect the effectiveness of subacute stroke rehabilitation. A quality comparison between muscle synergies computed with the conventional within-task peak/mean EMG normalization and our proposed method showed that the conventional was prone to activation amplitude overestimation and underestimation. The contributed method and findings help recapitulate and understand the post-stroke motor recovery process, which may facilitate developing more effective rehabilitation strategies for future stroke survivors.

## 1. Introduction

Virtually every country in the world is experiencing a shift in the age distribution of its population toward older ages (United Nations, 2019). The prevalence of age-related chronic diseases such as stroke is anticipated to increase steeply as the global population rapidly ages (James et al., 2018). Stroke has been the worldwide leading cause of disability and mortality (World Health Organization, 2019). Post-stroke patients suffer from significant motor deficiency and diminished independence in performing fundamental sit-to-stand (STS) movement (Cheng et al., 1998), which is the starting point of daily life and a reflection on one's quality of life. When transferring body momentum upwards from a sitting position, post-stroke patients are susceptible to falls, a well-known source of high injury severity and mortality (Cheng et al., 1998; Sterling et al., 2001). Repetitive and facilitated rehabilitation training on STS in addition to usual care can improve the patient's motor abilities (de Sousa et al., 2019).

At present, stroke rehabilitation demands evidence-based treatment strategies tailored to the needs of individual post-stroke patients (Hatem et al., 2016). Many studies investigated post-stroke STS muscle activities in hopes of discovering suitable rehabilitation strategies. It was found that patients exhibit distinctive muscle activation, such as delayed activation in tibialis anterior (Silva et al., 2013), premature activation in soleus (Cheng et al., 2004), prolonged duration of muscle activities (Chou et al., 2003), postponed activation peak timing of muscle synergy temporal patterns for hip raise (Yang et al., 2019), as well as diminished muscle strength (Jones, 2017).

Muscle activation amplitude and activation timing are two defining features regarding muscle activities. Muscle activity, detected by surface electromyography (EMG) devices placed on the skin and displayed in electromyogram, is a collective electrical signal acquired from activated muscle tissues (Raez et al., 2006). As a function of time, EMG signal is described by its amplitude and duration, reflecting both peripheral and central properties of the neuromuscular system (Farina et al., 2004). Depending on the muscle type, both muscle activation amplitude and activation timing may vary as mobility restores over time, and hence studies on both activation amplitude and timing are indispensable to an ampler understanding of whether and how the process of motor ability restoration would reflect in these biomechanical attributes.

Abnormal muscle activation timing features, such as delayed activation peak time and prolonged duration, may suggest that patients alter neural control strategies in executing movements. On the other hand, muscle activation amplitude is related to the magnitude of muscle tension and the maximum ability to produce muscle force (Vigotsky et al., 2018). As both muscle activation and muscle force depend on the number of active motor units, EMG amplitude can be applied to estimate muscle tension (Farina et al., 2004; Farina, 2006). EMG amplitude is also a valid measure to help interpret the contribution of muscle strength, facilitate diagnoses, and direct treatment strategies (Edgerton et al., 1996). Diminished amplitude of muscle activation patterns is an indicative factor of loss of independence and mobility impairment (Cheng et al., 2004; Lomaglio and Eng, 2005; Jones, 2017). However, isolated usage of EMG amplitude to explain the underlying neuromotor adaptation mechanism and infer outcomes in sports and rehabilitation medicine is prone to misinterpretations and should be avoided (Farina, 2006; Enoka and Duchateau, 2015; Del Vecchio et al., 2017; Vigotsky et al., 2018), although the popularity of its practice in research design has been surging since 1950 (Vigotsky et al., 2018). As a scaling factor, EMG amplitude alone does not account for muscle architectural properties or muscle contraction dynamics, such as muscle fiber length, physiological cross-sectional area, pennation angle, muscle contraction velocity, and passively generated muscle tension (Zajac, 1989; Hicks et al., 2015). When combined with musculoskeletal models integrated with these parameters, EMG data can facilitate estimating muscle tension, which is an informative and important factor often investigated in biomechanics research (Staudenmann et al., 2010; Hicks et al., 2015). To answer the question of whether and how muscle strength may change following neuromotor recovery in stroke rehabilitation, we avoid isolated interpretation of EMG data in this study by employing musculoskeletal modeling, in which discrete muscle is represented by the well-established Hill-type muscle model (Zajac, 1989) to capture necessary muscle properties and muscle-tendon dynamics (Staudenmann et al., 2010; Hicks et al., 2015; Vigotsky et al., 2018).

While EMG-informed analysis of muscle tension production using neuromusculoskeletal modeling is important for biomechanics questions (Hicks et al., 2015), muscle synergy analysis has been another useful way to study the complex mechanism underlying human movement. First proposed by Bernstein, muscle synergy hypothesis suggests that the human central nervous system controls modules of muscles in synergies to solve muscle redundancy and accomplish motor tasks (Bernstein, 1967). Although the ambiguity concerning whether muscle synergy can truly represent the neural strategy of motor control still remains (Tresch and Jarc, 2009; Kutch and Valero-Cuevas, 2012; Hirashima and Oya, 2015), numerous studies have implemented muscle synergy to infer motor deficiency in population with neurological disorders (Clark et al., 2010; Roh et al., 2013; Steele et al., 2015; Ellis et al., 2017; Mileti et al., 2020). Including our group's previous works, activation timing features of post-stroke muscle synergy were examined to simplify the complex control and coordinative recruitment of muscles, understand and translate indicative synergy time changes into more effective rehabilitation interventions for future stroke survivors (Yang et al., 2017, 2019; Kogami et al., 2018, 2021). It has been demonstrated that musculoskeletal modeling and simulation enhance muscle synergy analysis, which helps answer experimental questions in biomechanics and rehabilitation research (Steele et al., 2013; Hicks et al., 2015; Vigotsky et al., 2018).

Previous studies utilizing STS muscle activation features to indicate motor impairment and predict chances of motor recovery in the subacute stage focused on characteristics of activation timing, but they did not address activation amplitude (Prudente et al., 2013; Silva et al., 2013; Yang et al., 2019). Although Cheng et al. (2004) reported qualitative findings, such as post-stroke patients who tend to fall in STS have no or merely low-amplitude activation in tibialis anterior, it was not clear how activation amplitude respecting this fundamental movement would quantitatively reflect in stroke rehabilitation. The subacute stroke rehabilitation period (one to six months after stroke onset), wherein the most prominent recovery took place (Langhorne et al., 2011; Hatem et al., 2016), deserves more attention for studies on the patient's motor recovery progress. To better enable a longitudinal investigation into the patient's muscle activation changes during rehabilitation or an inter-subject comparisons, EMG normalization is usually applied (Besomi et al., 2020). Interpretations based on non-normalized EMG should be avoided if possible, and misinterpreted conclusions are often made if EMG data are not properly normalized before comparisons (Farina et al., 2004; Besomi et al., 2020). In our case, prior to studying the patient's muscle activation amplitude differences before and after rehabilitation, EMG normalization is necessary because EMG signal may be influenced by factors such as different skin conditions and electrode positions between measurement days (Besomi et al., 2020).

The most commonly used normalization technique by studies on muscle activation amplitude and muscle strength is normalization to the maximum voluntary contraction (MVC) (Raez et al., 2006; Besomi et al., 2020). MVC normalization may suit most non-disabled people, but it may be inapplicable to subacute stroke survivors who are unable to undertake MVC measurements or voluntarily activate muscles due to motor paralysis after the life-threatening cerebrovascular accident (Besomi et al., 2020).

In that case, prior studies with post-stroke patients accomplished normalization without utilizing MVC by adopting the in-task peak/mean EMG amplitude for normalization (Besomi et al., 2020), such as using within-trial peak EMG (Yang et al., 2019; Kogami et al., 2021), within-subject peak EMG across trials (Clark et al., 2010; Prudente et al., 2013), and peak EMG of the ensemble average (Cheng et al., 2004). Normalization utilizing the peak or mean EMG amplitude facilitates the examination and interpretation of EMG timing features, including peak timing, on/off timing of activities, and periods of inactivity, but it does not enable EMG amplitude comparisons (Cheng et al., 2004; Prudente et al., 2013; Yang et al., 2019; Besomi et al., 2020). Essentially, the method scales a large distribution of EMG data, taking the peak or mean EMG derived from trials as 100%, and removes the inherent differences in maximum EMG amplitude (Besomi et al., 2020). Despite the current consensus for experimental design involving EMG, there may be no normalization method available in some cases when participants have difficulties or cannot voluntarily activate a muscle (Besomi et al., 2020). Therefore, we aim to propose a novel normalization method that not only retains EMG amplitude differences for comparisons but also suits subacute stroke patients whose MVC cannot be obtained, in order to study the changes in muscle activation amplitude during recovery.

As analyses of muscle tension and muscle synergy can be complemented by musculoskeletal modeling and simulation (Steele et al., 2013; Hicks et al., 2015; Vigotsky et al., 2018), we sought to determine normalized muscle activation utilizing an originally proposed musculoskeletal model informed with experimental EMG and kinematics of post-stroke patients. Inverse dynamics is a common approach to calculating joint torques and muscle activation. However, simulated muscle activation often shows poor conformity to measured EMG (Hicks et al., 2015; Shuman et al., 2019). Forward dynamics, on the other hand, was widely used for muscle activation simulation in prior studies of human movement (Neptune et al., 2009; An et al., 2014, 2015; Hicks et al., 2015; Mehrabi et al., 2019). Muscle activation can be determined *via* optimization while accounting for co-contraction and muscle redundancy (Kutch and Valero-Cuevas, 2012; An et al., 2014, 2015; Trinler et al., 2018). However, assessments of simulated muscle activation against experimental EMG data have largely been qualitative rather than quantitative (Shuman et al., 2019). Nevertheless, various optimization algorithms have been proposed, including more customized ones often accompanied by highly personalized model design (Liu et al., 2008; Walter et al., 2014) and the commonly used static optimization (SO) by minimizing the squared error of muscle activation (An et al., 2014, 2015; Shuman et al., 2019; Wang et al., 2022).

For medical diagnosis and treatment, the most applicable and clinically relevant information is about the longitudinal outcome investigated serially by kinematic and kinetic measurements in conjunction with clinical assessments (Kwakkel et al., 2017; Vigotsky et al., 2018; Awad et al., 2020). EMG-informed conclusions made by researchers can easily become unsubstantiated due to the complexity of EMG data itself and insufficient longitudinal investigations (Vigotsky et al., 2018). Therefore, we aim to identify and quantify longitudinal changes in both muscle tension and muscle synergy to unravel the underlying biomechanical and neuromotor rehabilitation process within the post-stroke subacute period when patients are subjected to a greater likelihood of recovery. Due to the limitation of conventional EMG normalization methods, we propose a musculoskeletal model to determine normalized muscle activation through a combination of inverse dynamics joint torque calculation, EMG-informed forward dynamics simulation of joint torque and muscle activation, and an optimization algorithm to define muscle activation amplitude. Due to the multiple scopes of this research, we first evaluate muscle activation estimation results by the proposed normalization against results by traditional MVC normalization; next, we show the accuracy improvement of the proposed joint torque-based algorithm for musculoskeletal simulation compared to that of the most common SO based on squared muscle activation; finally, following a quality comparison between muscle synergies computed with our proposed method and the conventional peak EMG normalization in previous studies, we demonstrate the methodological impact on synergy structures and longitudinal changes in muscle tension and muscle synergy during subacute stroke rehabilitation to address our ultimate goal to provide new perspectives and independent reference data for future research on stroke rehabilitation. We also believe that modeling to recapitulate the process of functional recovery after motor dysfunctions is a promising approach to understanding hyper-adaptability in humans, the mechanism of adaptation to changes in the nervous and musculoskeletal systems (Eberle et al., 2021).

## 2. Methods

### 2.1. Musculoskeletal Modeling

The proposed approach realizes the biomechanical phenomenon of joint torques being generated by active and passive tension forces coactivated in skeletal muscles and the correlation between muscle activation and joint torques (Lomaglio and Eng, 2005; Vena et al., 2015; Jones, 2017). It was suggested that larger joint torques are reflected by muscles in greater activation. In this section, we present a novel method to normalize and scale a distribution of muscle activation signals based on joint torques; specifically, compute virtually normalized muscle activation using musculoskeletal models constructed based on the human body's anatomical, kinematic, and dynamic characteristics. First, joint torques are calculated from pre-defined and measured body kinematics in a four-link skeletal model by inverse dynamics principles (Section 2.1.1). Next, a musculoskeletal model incorporating the Hill-type muscle model (Zajac, 1989) is built for forward dynamics simulation and mapping of joint torques, in which muscle-tendon dynamics, anthropometry, and muscle activation are integrated besides body kinematics (Section 2.1.2). Lastly, muscle activation amplitude is determined by mathematical optimization to overcome muscle redundancy problems (Section 2.1.3).

#### 2.1.1. Skeletal Model and Inverse Dynamics

**Figure 1:**
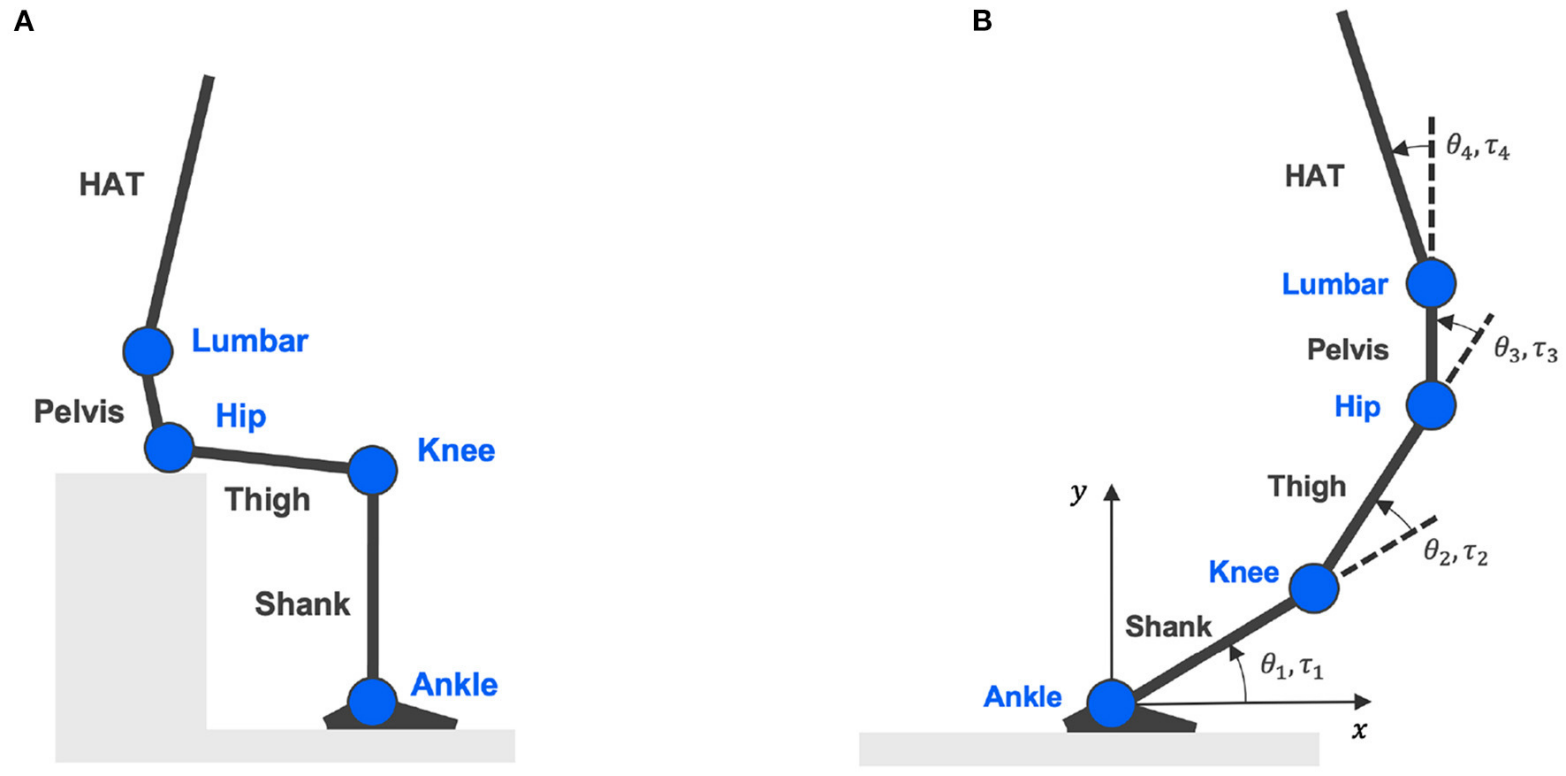
Four-link skeletal model. **(A)** Is the skeletal model representing body segments of shank, thigh, pelvis, and HAT (head, arm, and truck); connected by four joints: ankle, knee, hip, and lumbar. **(B)** Shows the definitions of joint angles and joint torques.

A four-link skeletal model was constructed to represent the entire human body in the sagittal plane with four segments and four joints to calculate joint torques from measured body kinematics (An et al., 2015). As in Figure 1A, each link specifies a segment of the human body as shank, thigh, pelvis, and HAT (head, arm, and truck). The four nodes connecting each segment indicate joints of the ankle, knee, hip, and lumbar. Definitions of joint angles and joint torques are shown in Figure 1B, where θ_*k*=1,2,3,4_ and τ_*k*=1,2,3,4_ symbolize joint angles and joint torques formed at the ankle, knee, hip, and lumbar, respectively.

Torques generated at each joint can be calculated given body kinematics and external forces using the following equation of motion (An et al., 2015):


(1) $$\begin{array}{l} \text{I}\left(\Theta ,\dot{\Theta }\right)\ddot{\Theta }+\text{H}\left(\Theta ,\dot{\Theta }\right)+\text{g}\left(\Theta \right)+\text{Ψ}\left(\Theta ,\dot{\Theta }\right)={\text{T}}_{\text{jnt}}+\Phi \left(\Theta ,\dot{\Theta }\right), \end{array}$$

where Θ is a vector representing joint angles θ_*k*=1,2,3,4_ calculated from measured joint positions at the ankle, knee, hip, and lumbar. Matrices $\text{I}\left(\Theta ,\dot{\Theta }\right)$, $\text{H}\left(\Theta ,\dot{\Theta }\right)$, **g**(Θ) indicate the moment of inertia of a segment, non-linear forces, and gravitational force, respectively. $\Phi \left(\Theta ,\dot{\Theta }\right)$ is a matrix of reaction forces at the feet and hip. $\text{Ψ}\left(\Theta ,\dot{\Theta }\right)$ is a matrix denoting the endured viscous resistance forces at each joint. Its magnitude is contingent on the type of joint at which the forces are received. Specified in Equation (2), resistance force $\text{Ψ}\left(\Theta ,\dot{\Theta }\right)$ is determined by joint angular velocity $\dot{\theta_{k}}$ at the ankle, knee, and hip joints (*k* = 1, 2, 3) (Davy and Audu, 1987); whereas $\text{Ψ}\left(\Theta ,\dot{\Theta }\right)$ is defined by the range of joint angle at the lumbar joint (*k* = 4) (Christophy et al., 2012).


(2) $$\begin{array}{l} \Psi (\Theta ,\dot{\Theta })=\{\begin{array}{ll} d_{k}\dot{\theta_{k}} & k=1,2,3\\ d_{k}^{\text{ext}}\theta_{k} & k=4,\theta_{k}>0.0314\\ d_{k}^{\text{flex}}\theta_{k} & k=4,\theta_{k}<-0.0314. \end{array} \end{array}$$

Lastly, **T**_jnt_ is the only unknown variable in Equation (1), denoting torques generated at each joint during STS. Given body kinematics and existing anatomical knowledge, joint torque **T**_jnt_ can be calculated following the inverse dynamics principle.

#### 2.1.2. Forward Dynamics Simulation

**Figure 2:**
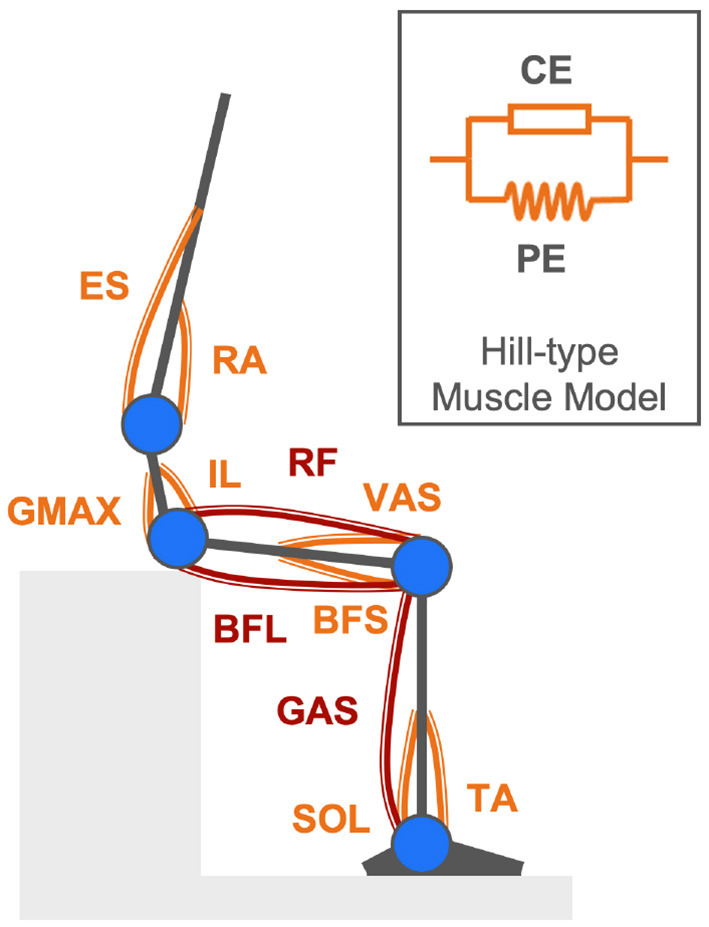
Musculoskeletal model comprising eight uniarticular muscles (orange components) and three bi-articular muscles (maroon components) described by the Hill-type muscle model for rendering the human body STS movement in forward dynamics simulation.

While joint torques are calculated given body kinematics with the four-link skeletal model and inverse dynamics, joint torques are also simulated by forward dynamics (An et al., 2015). The proposed musculoskeletal model comprising 11 uniarticular and bi-articular muscles essential for rendering the human body STS movement by forward dynamics is shown in Figure 2. In this model, joint torques are supposed to be generated by net forces in contracted and passively activated muscles. The Hill-type muscle model is applied to describe individual muscle force production in two elements: contractile element (CE) and parallel element (PE) (Zajac, 1989). Names of the 11 modeled muscles are Tibialis Anterior (TA), Soleus (SOL), Gastrocnemius (GAS), Rectus Femoris (RF), Vastus Lateralis (VAS), Biceps Femoris Long Head (BFL), Biceps Femoris Short Head (BFS), Gluteus Maximus (GMAX), Rectus Abdominis (RA), Erector Spinae (ES), and Iliopsoas (IL).

The simulated joint torque **τ**_jnt_ is represented by a vector of torques τ_*k*=1,2,3,4_ around the ankle, knee, hip, and lumbar joints, as in Equation (3). Torque τ_*k*_ at individual joints is a net quantity of torques generated by each associated muscle exerting tension *F*_*i*_ about the joint center at an equivalent distance of the anatomical muscle moment arm *r*_*ki*_, as in Equation (4). *r*_*ki*_ designates the moment arm of the *i*th muscle (*i* = 1, ..., 11) attached to the *k*th joint (*k* = 1, 2, 3, 4). According to the Hill-type muscle model, muscle tension *F*_*i*_ is a combination of the actively generated force $F_{i}^{\text{CE}}$ by the contractile element (CE) and a passive force $F_{i}^{\text{PE}}$ contributed by the parallel element (PE), as in Equation (5).


(3) $$\begin{array}{l} \tau_{\text{jnt}}={\left[\begin{array}{cccc} \tau_{1} & \tau_{2} & \tau_{3} & \tau_{4} \end{array}\right]}^{\text{T}}, \end{array}$$

(4) $$\begin{array}{l} \tau_{k}=\overset{4}{\underset{k=1}{\sum }}\overset{11}{\underset{i=1}{\sum }}r_{ki}F_{i}, \end{array}$$

(5) $$\begin{array}{l} F_{i}=F_{i}^{\text{CE}}+F_{i}^{\text{PE}}. \end{array}$$

The actively generated contraction force $F_{i}^{\text{CE}}$ in the *i*th muscle is determined by the *i*th muscle's isometric maximum muscle force $F_{i}^{\text{max}}$, force-length relationship *f*_fl_, force-velocity relationship *f*_fv_, and normalized muscle activation ${\hat{m}}_{i}$, as in Equation (6). The normalized muscle activation ${\hat{m}}_{i}$ is unknown and will be solved following computations detailed in the next Section 2.1.3. The two time-varying dynamic muscular properties of force-length relationship *f*_fl_ and force-velocity relationship *f*_fv_ are defined in Equations (7), (8), respectively (Hatze, 1977; Ogihara and Yamazaki, 2001), where *f*_fl_ is a function of the normalized *i*th muscle length to its optimal muscle length, denoted by ${\hat{l}}_{i}$, and *f*_fv_ is a function of the normalized *i*th muscle contraction velocity to the maximum muscle contraction velocity, denoted by ${\hat{v}}_{i}$.


(6) $$\begin{array}{l} F_{i}^{\text{CE}}=F_{i}^{\text{max}}f_{\text{fl}}f_{\text{fv}}{\hat{m}}_{i}, \end{array}$$

(7) $$\begin{array}{l} f_{\text{fl}}=\text{exp}\left(-{\left(\hat{l_{i}}-1\right)}^{2}\right), \end{array}$$

(8) $$\begin{array}{l} f_{\text{fv}}=1+\text{tanh}\left(3{\hat{v}}_{i}\right). \end{array}$$

Additionally, the passive force $F_{i}^{\text{PE}}$ in Equation (5) by the parallel element (PE) is produced when a muscle stretches and exceeds its optimal muscle fiber length. Once generated, this passive force is related to the normalized muscle length ${\hat{l}}_{i}$ and the isometric maximum muscle force $F_{i}^{\text{max}}$, as in Equation (9).


(9) $$\begin{array}{l} F_{i}^{\text{PE}}=\{\begin{array}{ll} 0 & {\hat{l}}_{i}<1.0\\ F_{i}^{\text{max}}\frac{e^{10(\hat{l}-1)}}{e^{5}} & 1.0\leq {\hat{l}}_{i}\leq \text{1}.\text{5}\\ F_{i}^{\text{max}} & 1.5<{\hat{l}}_{i}. \end{array} \end{array}$$

#### 2.1.3. Muscle Activation Calculation

The proposed musculoskeletal model incorporates several biarticular muscles, which induces the muscle redundancy problem as there are more muscles than mechanical degrees of freedom (Kutch and Valero-Cuevas, 2011). In forward dynamics simulation, the computation of torques produced by 11 types of muscles at four joint positions are governed by Equation (4); subsequently, creating an underdetermined system of equations with 11 unknowns and four equations. The underdetermined system of equations is solved by optimization with additional constraints. The goal is to enable the proposed musculoskeletal model to simulate movements that resemble the actual STS movements performed in reality.

All the unknown muscle activation ${\hat{m}}_{i}$ from Equation (6) is determined by finding the optimal activation that can simulate joint torques equivalent to the torques calculated by inverse dynamics from body kinematics to replicate the observed STS movement. Optimization is implemented under the constraint that the simulated muscle activation ${\hat{m}}_{i}$ and the measured muscle activation preserve a perfect positive correlation throughout the motion progress; meanwhile, subjected to an objective function defined as to minimize the errors between the extrema of simulated joint torques **τ**_jnt_ and joint torques **T**_jnt_ calculated from body kinematics, as in Equation (10).


(10) $$\begin{array}{l} Z=||T_{jnt}^{\max}-\tau_{\text{jnt}}^{\text{max}}|{|}^{2}+||T_{jnt}^{\min}-\tau_{\text{jnt}}^{\text{min}}|{|}^{2}. \end{array}$$

### 2.2. Muscle Synergy Model

Muscle synergy theory modularizes the complex control of individual muscles into a limited number of synchronized muscle activation to explain each different type of human body movement (Ivanenko et al., 2004; Clark et al., 2010). Human STS movement can be explained by four synergies for healthy subjects and post-stroke patients (Yang et al., 2017), with each synergy corresponding to the phase of lumbar flexion, hip raise, body extension, and posture control in STS (Schenkman et al., 1990). By analyzing muscle synergy patterns, post-stroke STS accomplished by the redundant human body can be easily understood from the perspective of muscle coordination in motor control units. The time-dependent muscle activation is expressed as a linear summation of spatiotemporal patterns as in Equation (11), where matrices **M**, **W**, and **C** indicate muscle activation, spatial pattern, and temporal pattern, respectively.


(11) $$\begin{array}{l} \text{M}=\text{W}\text{C}. \end{array}$$

The *n* × *t*_max_ matrix **M** of muscle activation comprises time-varying muscle activation vectors **m**_*i*(*i*=1,2,...,*n*)_ which represent muscle activation in *n* different muscles at time 1 < *t* < *t*_max_.

The *n* × *N* matrix **W** denotes muscle synergy spatial patterns in which relative activation levels of muscles are defined. Each column in the **W** matrix refers to one of the *N* different numbers of synergies **w**_*j*(*j*=1,2,...,*N*)_. Vector **w**_*j*(*j*=1,2,...,*N*)_ represents the relative activation level of the *i*th muscle (*i* = 1, 2, ..., *n*) in the *j*th muscle synergy (*j*=1,2,...,*N*).

The time-dependent *N* × *t*_max_ matrix **C** of temporal patterns defines the weighting coefficient of each of the *N* different muscle synergies in a vector **c**_*j*(*j*=1,2,...,*N*)_. Vector **c**_*j*(*j*=1,2,...,*N*)_ is a time-varying scaling factor of the corresponding spatial pattern **w**_*j*(*j*=1,2,...,*N*)_ at time 1 < *t* < *t*_max_.

**Figure 3:**
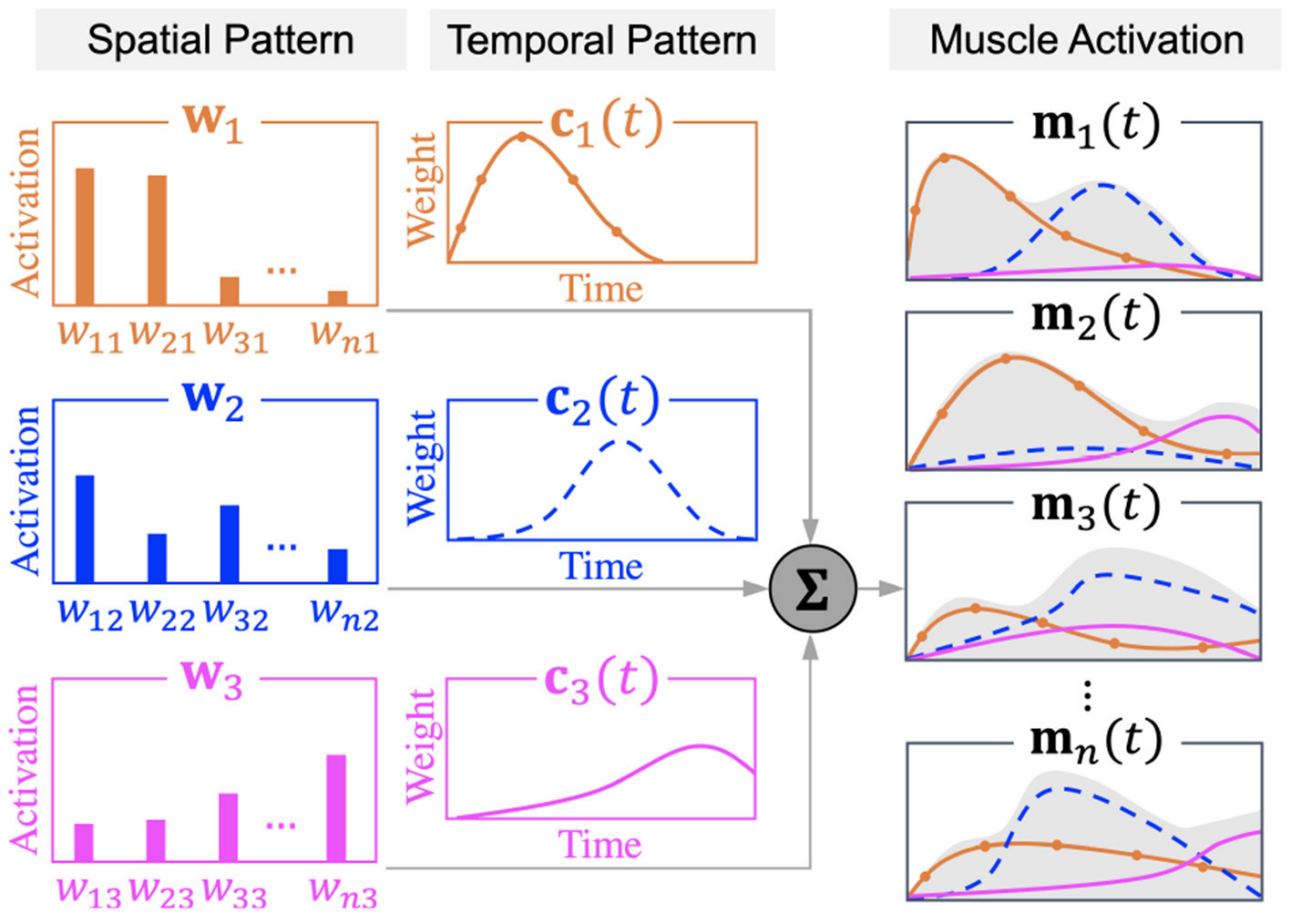
Muscle synergy model.

Figure 3 shows the schematic design of a muscle synergy model that exemplifies the case of *n* muscles' activation expressed as the linear summation of spatial patterns (**w**_1,2,3_) and temporal patterns (**c**_1,2,3_) of three muscle synergies. Activation in each of the *n* muscles is depicted by the gray area under the curve.

For the extraction of muscle synergy patterns **W** and **C** from muscle activation **M**, non-negative matrix factorization (NNMF) was used (Lee and Seung, 1999). Muscle synergies were extracted from each trial of each subject (Ivanenko et al., 2005; Oliveira et al., 2014; Yang et al., 2017; Kogami et al., 2021).

### 2.3. Measurement Experiment

#### 2.3.1. Subjects

Eight healthy male adults (age 23.8 ± 2.6) and four post-stroke patients (age 55.0 ± 4.8) participated in a series of measurement experiments.

Each healthy participant performed 15 trials of STS movement by standing up at a self-paced speed from a seat adjusted to the height of the person's knee. Measurements of maximum voluntary contraction (MVC) were conducted with healthy participants to obtain ground truth data for the validation and assessment of the proposed method.

Post-stroke patients were invited to serial STS measurements during their subacute rehabilitation stay (137 ± 22 days) in the hospital in order to compare and investigate individual patient's biomechanical changes at different times in the subacute stage. Two patients finished two measurements, and the other two each finished three and four measurements. On each measurement day, the patient was asked to do ten STS trials without assistance at a self-paced speed from a seat adjusted to the height of the knee. Patients' improvements in Fugl-Meyer Assessment (FMA) clinical score of the lower limb motor recovery over their inpatient subacute rehabilitation were +13, +8, +3, and 0 points, respectively.

All the healthy and patient participants were asked to remain their feet still at a comfortable position throughout measurement trials.

Informed consent was obtained from healthy participants and patients before the experiment, which was approved, respectively, by the Institute Review Board of The University of Tokyo and the Institute Review Board of Morinomiya Hospital, Osaka, Japan.

#### 2.3.2. Measurements

**Figure 4:**
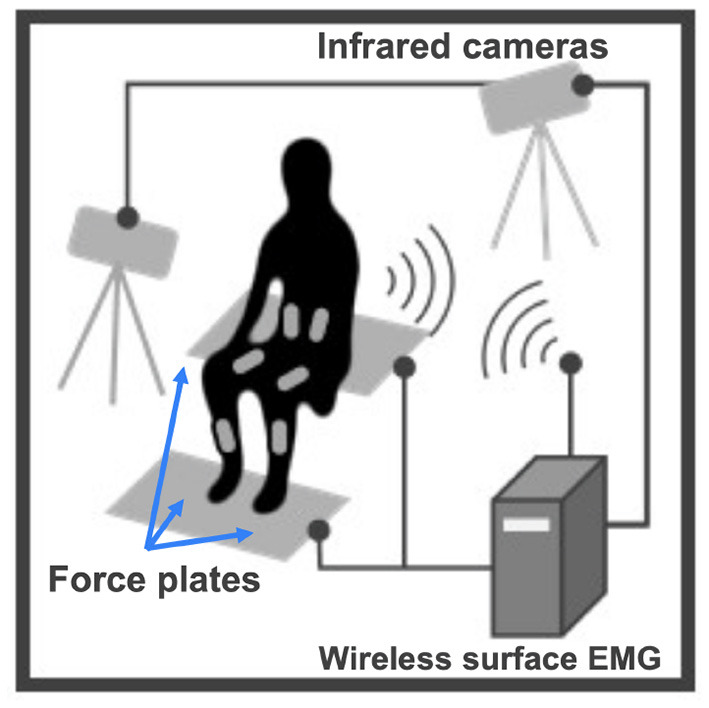
STS measurement experiment setup.

STS experiment setup is shown in Figure 4. An optical motion capture system (Motion Analysis Corp.), including 14 infrared cameras, was used to record body kinematics at 100 Hz. Body kinematics data, such as joint positions, were used to calculate joint angles in SIMM (Musculographics, Inc.). Three separate force plates (TechGihan Corp.) were set under the hip and each foot to measure the reaction forces at 2,000 Hz. The seat-off moment was marked when the vertical force recorded under the hip dropped below 10 N. Reaction force data were low-pass filtered at 20 Hz. Wireless surface Electromyography (EMG) sensors (Cometa Corp.) were directly placed on the participant's skin to record muscle activity. Surface EMG sensors were placed at ten types of uniarticular and biarticular muscles in the upper trunk and lower limbs to obtain muscle activation signals at 1,000 Hz for healthy subjects and 2,000 Hz for post-stroke patients. Each recorded muscle contributes to accomplishing STS movement by either flexion or extension at one or two joints of the ankle, knee, hip, and lumbar. Measured muscles are the same ones considered in the proposed musculoskeletal model (TA, SOL, GAS, RF, VAS, BFL, BFS, GMAX, RA, ES), except for Iliopsoas (IL), since IL is an inner hip flexion muscle and its activation cannot be measured with surface EMG sensors. Surface EMG electrodes locations were determined by the point on a line between two anatomical landmarks of individual muscles, according to the European SENIAM recommendations (Hermens et al., 2000; Blanc and Dimanico, 2010). Muscle activation signals were band-pass filtered with a zero-lag fourth-order Butterworth filter of 40–400 Hz and rectified with a fourth-order low-pass Butterworth filter at 4 Hz (Clark et al., 2010; Gizzi et al., 2011). All EMG data were recorded continuously throughout measurement trials with the participant. Each STS trial was extracted from the entire process according to the seat-off time such that one STS trial is a discrete 3-second interval, with one second before the seat-off moment and two seconds elapsed after seat-off.

Additionally, in the MVC activation measurement with healthy subjects, an experimenter applied resistive forces by hand in different directions to the participant's joints at the ankle, knee, hip, and lumbar while the participant voluntarily exerted a maximum force by muscle contraction to push or pull against the applied force. EMG signals were recorded for the same types of muscles (TA, SOL, GAS, RF, VAS, BFL, BFS, GMAX, RA, and ES). EMG signals collected in the MVC measurement were processed in the same way as those collected from STS measurements. For each type of muscle, the MVC activation, to which all the EMG data of healthy subjects were normalized, was determined by the maximum muscle activation value during the participant's measurement.

## 3. Results

### 3.1. Muscle Activation Estimation Accuracy

Simulation results were evaluated against the measured muscle activation data from eight healthy subjects to validate the proposed method. All courses of computation were done in MathWorks MATLAB (2021). Two factors were evaluated between the simulated and measured muscle activation, namely, Pearson's correlation coefficient (Pearson's *r*) and root-mean-square error (*RMSE*). Table 1 shows within-muscle Pearson's *r* of the ensemble average across subjects to demonstrate the quality of conformity between simulation and the observed MVC-normalized muscle activation (Cheung et al., 2005; Staudenmann et al., 2010; Laine et al., 2021). All muscles (TA, SOL, GAS, RF, VAS, BFL, BFS, GMAX, RA, and ES) achieved a Pearson's *r* = 1 (Mukaka, 2012) throughout the motion progress due to the constrained optimization conditions. *RMSE* informs the within-muscle amplitude error between the peak values in simulation and the observed reference. Table 1 also compares the simulation performance of our proposed joint torque-based algorithm with that of the commonly used SO algorithm based on squared muscle activation (An et al., 2014, 2015; Shuman et al., 2019; Wang et al., 2022). Both enhancements in correlation and reduction of amplitude error are reported as a percentage quantity, showing the better performance of the proposed method. Due to the constrained correlation (*r* = 1) between simulated muscle activation and measured EMG, the onset and offset timing in simulations and experiments also aligned perfectly. Additionally, the percentage relative error (%*RE*) (Kat and Els, 2012) between the simulated and calculated joint torque maxima in motion progress was 8.9 ± 5.9%. The knee joint, especially, scored the highest accuracy with a minimal %*RE* of 0.36 ± 0.83%. Since the activation of Iliopsoas (IL), an inner hip flexor, could not be measured using surface EMG sensors in the experiment, its simulation accuracy was not verified. Therefore, barring IL muscle, all the other muscles of post-stroke cases will be scrutinized for activation changes over the subacute rehabilitation period. The reconstruction quality with four synergies obtained using trial-by-trial extraction of muscle synergy was 94.2 ± 3.4%, overall satisfied the threshold of 90% (Cheung et al., 2005).

**Table 1:** Muscle activation simulation results evaluated against measured MVC-normalized EMG from healthy subjects.

	**Pearson's** **r**			**RMSE**		
**Muscle name**	**SO**	**Proposed**	**Improvement**	**SO**	**Proposed**	**Improvement**
TA	0.669	1.00	49.5%	0.0691	0.0617	10.7%
GAS	0.469	1.00	113%	0.0253	0.0167	33.9%
SOL	0.604	1.00	65.6%	0.0335	0.0313	6.46%
RF	0.924	1.00	8.20%	0.0657	0.0274	58.3%
VAS	0.849	1.00	17.8%	0.0581	0.0266	54.2%
BFL	0.826	1.00	21.0%	0.0500	0.0266	46.7%
BFS	0.964	1.00	3.80%	0.0617	0.0226	63.4%
GMAX	0.982	1.00	1.90%	0.0698	0.0551	21.0%
RA	0.782	1.00	28.0%	0.1005	0.0865	14.0%
ES	0.778	1.00	28.5%	0.0279	0.0241	13.8%

*With reference to MVC-normalized EMG, an inter-method simulation accuracy comparison was done between the proposed joint torque-based algorithm and SO based on muscle activation. Muscle activation profile conformity and peak amplitude agreement are evaluated and compared by Pearson's correlation (−1 ≤ r ≤ 1) and root-mean-square error (RMSE), respectively, between the proposed and SO algorithms. Within-muscle simulation accuracy improvements by the proposed algorithm are reported as percentages*.

### 3.2. Post-stroke Muscle Activation Amplitude-Related Features

As muscle activation amplitude is related to the magnitude of muscle tension and the level of muscle synergy, this study examined activation amplitude-related features for post-stroke patients, including maximum muscle tension and peak level of muscle synergy. The following Sections 3.2.1 and 3.2.2 show results pertaining to the progression of patients' muscle tension and muscle synergy during the subacute rehabilitation period. Courses of computation were completed in MathWorks MATLAB (2021). Statistical significance of changes between different measurement days was decided by the Wilcoxon rank-sum test for patients with two times of measurements (Rosner et al., 2006) and by the Kruskal–Wallis test (one-way analysis of variance on ranks) for patients who completed three or more times of measurements (McDonald, 2014).

#### 3.2.1. Maximum Muscle Tension

After patients' STS muscle activation amplitude values were determined and virtually normalized based on joint torques by the proposed method, tension force generated in each muscle was calculated by Equations (5), (6). Regarding the intra-subject changes on the paretic side among four patients, significant increases (*p* < 0.001) to various extents were found in maximum muscle tension and peak muscle activation amplitude. RF and VAS are the only two muscles that did not show any significant peak tension reduction after rehabilitation in all patients. RF is a biarticular muscle acting as a knee extensor and hip flexor, whereas VAS is a uniarticular muscle acting as a knee extensor.

As in Table 2, the number of muscles that yielded significant increases in maximum tension was the greatest for the patient with the greatest motor recovery among the four. The patient with +13 points in FMA was detected to have eight muscles (TA, GAS, SOL, RF, VAS, BFS, RA, and ES) experiencing tension increases, comparing to two, four, and three muscles demonstrating tension increase in the other three patients with +8, +3, and +0 in FMA, respectively. Likewise, the number of muscles showing significant tension decreases after rehabilitation was the least in the most improved patient (FMA +13), with only two muscles (BFL and GMAX).

**Table 2:** Compare muscle tension results from post-stroke cases: changes in maximum muscle tension over the subacute rehabilitation period of four patients with +13, +8, +3, and +0 points in the Fugl-Meyer Assessment (FMA) of motor recovery.

	**Max muscle tension percentage change [%]**			
**Muscle name**	**Case: FMA +13**	**Case: FMA +8**	**Case: FMA +3**	**Case: FMA +0**
TA	892^*^	1.66^*^	−50.6^*^	−0.402
GAS	62.6^*^	−58.6^*^	−1.30	−5.32^*^
SOL	12.6^*^	−17.3^*^	31.8^*^	−33.6^*^
RF	111^*^	−0.272	215^*^	49.0^*^
VAS	19.1^*^	−2.29	0.0553	−2.00
BFL	−11.5^*^	−2.89^*^	102^*^	4.59^*^
BFS	592^*^	−2.60^*^	−35.1^*^	−49.3^*^
GMAX	−27.0^*^	−3.80	−58.7^*^	19.9^*^
RA	113^*^	57.9^*^	131^*^	−31.2^*^
ES	90.2^*^	1.30	−7.41^*^	−7.04^*^

* *Change with statistical significance, p < 0.001*.

As for the patients with more than two measurements, it was noted that the greatest peak muscle tension did not necessarily disclose on the last measurement day.

#### 3.2.2. Muscle Synergy Progression

With normalized muscle activation, muscle synergy spatial and temporal patterns were determined by NNMF, and the amplitude of synergy patterns became appropriate for comparisons. For all patients, their spatial patterns appeared similar over the subacute rehabilitation. The dominant muscles with the highest relative activation in spatial patterns in each muscle synergy were in line with the ones found by Yang et al. (2017). The peak level of patients' temporal patterns, however, exhibited some variations in rehabilitation. Summarized in Table 3, the patient with the greatest improvement in motor recovery (FMA +13) showed significant peak level increases (*p* < 0.05) in temporal patterns of all four muscle synergies; respectively, by 88.3, 152, 134, and 65.0%. In comparison, the other patients had fewer temporal patterns revealing peak level increases. The patient with no improvement in FMA only had an amplitude decrease in the temporal pattern of Synergy 1 (body flexion) by 15.9% after 154 days, while the patient's other temporal patterns of Synergy 2 (hip raise), Synergy 3 (body extension), and Synergy 4 (posture control) were found with no significant amplitude changes over time.

**Table 3:** Temporal synergy patterns progression in subacute rehabilitation.

	**Peak change in temporal patterns [%]**		
	**Case: FMA +13**	**Case: FMA +3**	**Case: FMA +0**
Synergy 1	88.3^*^	61.0^*^	−15.9^*^
Synergy 2	152^*^	−38.8^*^	9.27
Synergy 3	134^*^	46.2	15.9
Synergy 4	65.0^*^	35.5^*^	−4.01

*Results of three subacute stroke patients with +13, +3, and +0 points in the Fugl-Meyer Assessment (FMA) of motor recovery*.

* *Change with statistical significance, p < 0.05*.

**Figure 5:**
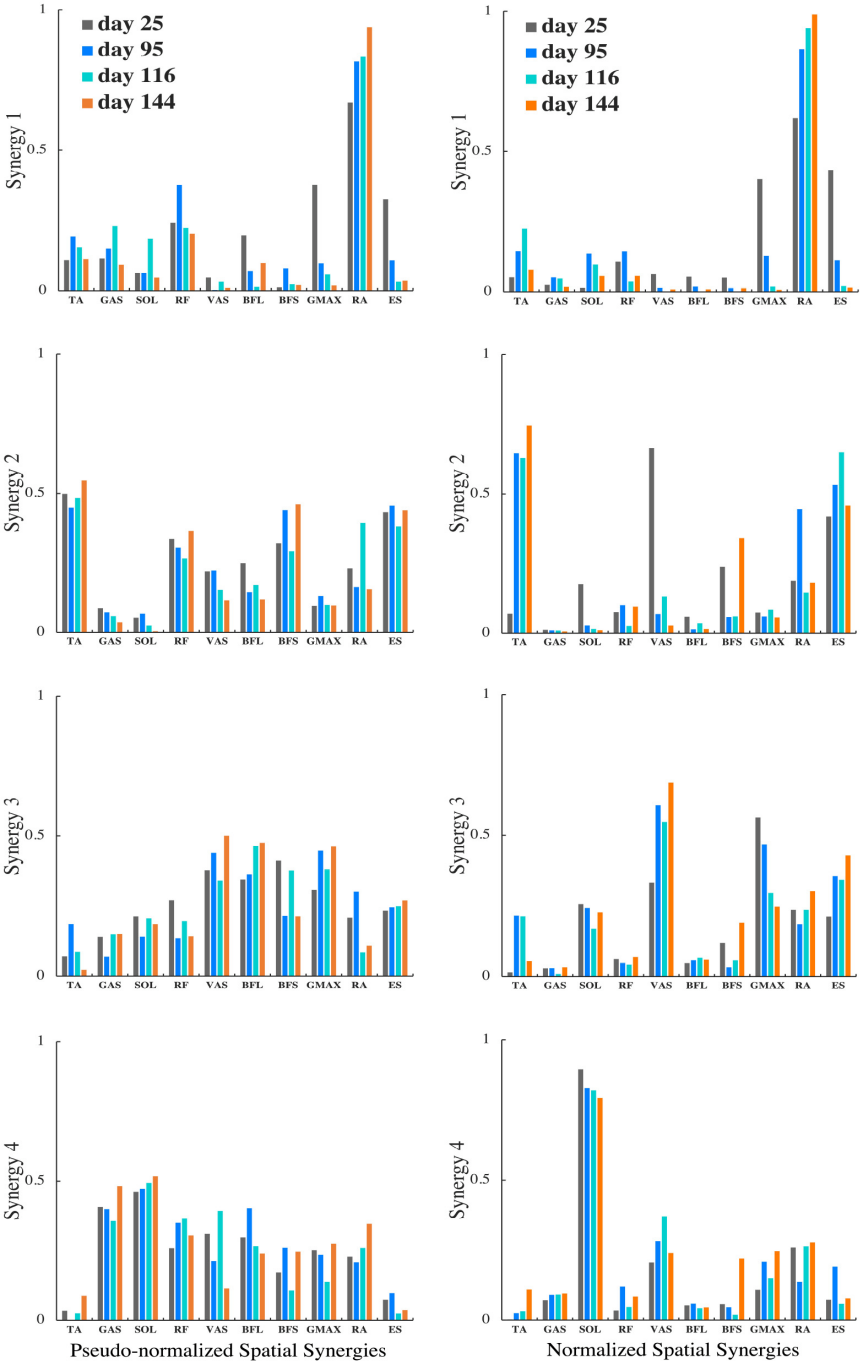
Muscle synergy spatial patterns of the representative patient with +13 points of improvement in FMA score on the 25th, 95th, 116th, and 144th day after initial stroke onset. The left column shows spatial patterns of each synergy normalized by within-subject peak EMG, noted as pseudo-normalized for comparison. The right column shows spatial patterns of each synergy normalized by proposed method. Vertical axis indicates relative muscle activation levels ranging from 0 to 1. Horizontal axis presents muscle names.

**Figure 6:**
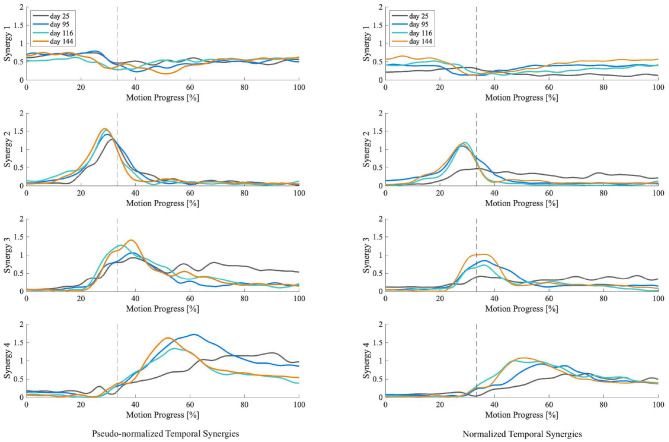
Muscle synergy temporal patterns of the representative patient with +13 points of improvement in FMA score on the 25th, 95th, 116th, and 144th day after initial stroke onset. The left column shows temporal patterns of each synergy normalized by within-subject peak EMG, noted as pseudo-normalized for comparison. The right column shows temporal patterns of each synergy normalized by proposed method. Vertical axis indicates the weighting coefficients. Horizontal axis is STS motion progress expressed in percentage. Dotted vertical lines represent the seat-off time.

For the patient with the greatest improvement in FMA of motor recovery, both amplitude and timing of temporal patterns showed distinctive changes throughout the subacute rehabilitation. Figure 5 shows the representative subject's normalized muscle synergy spatial patterns and their progression over 144 days of inpatient rehabilitation. Spatial patterns calculated with the conventional within-subject peak EMG normalization are also presented, noted as pseudo-normalized for comparison. Similarly, temporal patterns obtained by the proposed method and by the conventional within-subject peak EMG normalization can both be found in Figure 6.

## 4. Discussion

This study proposed a novel method to calculate muscle activation and normalize it based on joint torques. Upon validating the method with eight healthy young subjects, we applied the proposed approach with four subacute stroke patients to investigate their muscle activation amplitude-related biomechanical features such as muscle tension and muscle synergy level. Like in most cases, uncertainties and limitations of generic-scaled musculoskeletal modeling remained in this study, especially in analyses with post-stroke patients (Hicks et al., 2015; Kainz et al., 2021). Nevertheless, quantitative results found may hint that maximum muscle tension and activation level of muscle synergy temporal patterns could reflect the effectiveness of subacute stroke rehabilitation.

### 4.1. Method Validation

The proposed EMG-informed generic-scaled musculoskeletal model that estimates muscle tension in the Hill-type muscle model was compared to the existing approach of previous relevant studies as recommended by Hicks et al. (2015) to validate its performance. With reference to the MVC-normalized experimental muscle activation as a virtual ground truth, we compared simulation accuracy of the proposed joint torque-based method with the commonly used SO based on squared muscle activation. Shown in Table 1, the proposed method performed better across muscle types and estimated STS muscle activation more accurately; overall, with enhanced muscle activation profile conformity (greater Pearson's *r*) and reduced error in muscle activation peak amplitude (smaller *RMSE*). Moreover, in contrast to conventional within-subject peak normalization (Cheng et al., 2004; Silva et al., 2013; Yang et al., 2019), the normalized peak muscle activation in different muscles and subjects were no longer an indiscriminate value of 100%. Muscle activation profiles estimated in forward dynamics simulation and EMG curves measured in experiments are similar, and their critical onset and offset timings also perfectly agree (Hicks et al., 2015).

In this study, anthropometry parameters were carefully retrieved from past studies based on muscle-tendon data derived from human cadavers and MRI-based measurements of multiple subjects (Riener and Fuhr, 1998; Ward et al., 2009; Arnold et al., 2010). Muscle tension estimated by the Hill-type muscle model may be sensitive to parameters such as force-length relationship and isometric maximum muscle force (Scovil and Ronsky, 2006). Muscle moment arms and muscle geometry may also vary between different individuals, especially when pathological conditions are involved (Scheys et al., 2008; Kainz et al., 2021). These parameters, to which estimated muscle tension may be sensitive, were generalized between healthy and post-stroke groups in this study.

However, in our model, muscle tension was generated throughout the STS trial to meet the goal by producing the same amount of joint torques calculated from kinematics. Scovil and Ronsky (2006) found that muscle forces generated to track a specified trajectory or meet movement goals were less sensitive to muscle model parameters. Moreover, using EMG data collected from patients with neurological disorders to estimate muscle forces in the Hill-type model can overcome the limitation imposed by models assuming an identical neuromuscular control strategy between individuals (Lloyd and Besier, 2003; Hoang et al., 2018). Therefore, our generalized parameters may have less impact on simulations in the healthy group with which the model was initially validated. Meantime, we suggest that current muscle tension results of the neurologically impaired stroke group may be interpreted with caution due to the modeling limitation and our current small size of patients. Nevertheless, the current method demonstrated its ability to estimate muscle activation with markedly reduced errors between MVC-normalized EMG and perfectly aligned on/off timings with measured EMG from both healthy and post-stroke subjects.

To investigate the impact of normalization on muscle synergy analysis, we compared synergy progression results (over 144 days) normalized by the proposed method and those by the conventional peak EMG normalization utilized in previous studies (Cheng et al., 2004; Clark et al., 2010; Prudente et al., 2013; Yang et al., 2017, 2019; Kogami et al., 2018, 2021). As in Figure 5, in comparison, when muscle activation was normalized to within-subject peak activation (noted as pseudo-normalized in figures) prior to NNMF, spatial activation in dominant muscles in synergies are underestimated (e.g., RA in Synergy 1, TA in Synergy 2, VAS and ES in Synergy 3, SOL in Synergy 4), whereas the subordinate muscle contributors are prone to overestimation. In Figure 6, we presented the corresponding temporal pattern progression (over 144 days) normalized by the proposed method in tandem with that by the traditional peak EMG normalization. With the pseudo-normalized temporal patterns, differences in peak levels of temporal synergy are more subtle. The less evident amplitude changes in temporal and spatial patterns may be the consequence of the fact that within-subject peak EMG normalization indiscriminately scales peak activation in different muscles to 100%, which removes inherent peak activation differences and induces amplitude overestimation or underestimation. Conversely, peak timings of temporal synergy are very similar with both methods, which is favorable, as the peak EMG normalization has been broadly applied by past studies on synergy timing. It demonstrates that the proposed method can be useful for clarifying activation levels, as well as synergy timing features. The synergy activation timing agreement can be attributed to the perfect covariation and high conformity of our simulated muscle activation with experimental EMG. Lastly, we compared muscle synergy reconstruction quality by the proposed joint torque-based normalization with that by the within-subject peak EMG normalization reported previously in studies that had similar experimental design; on average, around 94% by the proposed and around 88% by peak EMG (Yang et al., 2017; Kogami et al., 2021).

### 4.2. Rehabilitation of Muscle Tension

Although the model's sensitivity and uncertainty may be affected by muscle geometry and pathology, muscle tension changes with regard to patients' sensorimotor functioning (i.e., FMA scores) found in this study can still provide new perspectives in answering if and how activation amplitude-related features would reflect in subacute stroke rehabilitation. Our results can be a reference for future studies on muscle tension with subacute stroke patients and help expand the pool of available independent data (Hicks et al., 2015).

Shown in Table 2, the difference between patients in the number of muscles yielding increased muscle tension after rehabilitation may indicate a correlation between the degree of mobility restoration and the capacity of muscle force production in lower limbs of subacute stroke patients. A similar difference in the number of muscles showing significant activation amplitude increases was also found between the sampled patients with various degrees of motor recovery. As past studies found that chronic stroke patients showed significant gains in isometric muscle strength and assessment scores of motor performance in upper limb muscles after receiving rehabilitation training (Lum et al., 2002), findings of this study may imply that the likelihood of seeing muscle activation increases on the paretic side is higher for subacute stroke patients with notable improvement in lower limb FMA. The greater number of muscles demonstrating diminished activation amplitude and tension in less-improved patients could suggest that patients who do not really recover in subacute rehabilitation may develop altered muscle activation strategies in accomplishing STS. One hypothesis may be that patients who improve poorly in the subacute stage tend to shun the usage or reliance of their paretic side; instead, they consciously or subconsciously rely more on the non-paretic side for training, which eventually resulting in motor compensation from the more capable side rather than true motor recovery on the paretic side.

The most improved patient (FMA +13) showed some exclusive progress with peak muscle tension, which other less-improved patients did not experience, such as increased tension in the ankle plantar-flexor and knee flexor GAS, knee extensor VAS, and lumbar extensor ES. On top of that, the most improved patient, who also happened to be the only one showing significant increases in ankle joint torque after rehabilitation, gained a phenomenal growth of peak muscle tension in the ankle dorsiflexor TA by 892%. Similar drastic changes were found with the patient's muscle activation amplitude as well. Besides, a small yet significant increase in TA's tension was also found with the second best-improved case (FMA +8). According to qualitative findings in the past, post-stroke patients with a greater burden on motor function and a higher chance to collapse in STS exhibited no or a merely perceptible low-amplitude activity in their TA muscles (Cheng et al., 2004). In this study, as patients with relatively higher FMA score improvements significantly enlarged both activation and tension in their TA muscles, it may suggest an important contributing factor to their outstanding rehabilitation, after which time their enhanced TA muscles mitigated the risk of collapsing.

As for the case of FMA +0, suggesting little to no recovery in terms of motor functioning, balance, sensation, and joint functioning after rehabilitation (Fugl-Meyer et al., 1975), the patient revealed significant increases in hip joint torque and the hip extensor GMAX's peak tension and activation amplitude after rehabilitation, which was something not observed in other patients with some degree of motor recovery. It could hint that patients with more restored motor ability do not employ their hips in STS the same way as those with less motor recovery. Furthermore, this patient showed a peak tension decrease in the lumbar flexor RA, whereas the other patients with FMA improvement returned with considerable tension increases in RA. The distinguished improvement in RA may indicate a better upper body momentum generation in STS with the better-improved cases. These hypotheses may be affirmed with a larger pool of subacute stroke patients in future studies.

This study also confirmed that increased muscle tension does not necessarily correspond to increased muscle activation amplitude and vice versa (Vigotsky et al., 2018), as EMG analysis does not encompass muscle geometry or muscle contraction dynamics. For future rehabilitation evaluation, we also suggest avoiding isolated usage of EMG data and encourage analyses of both EMG and muscle tension to study neuromotor recovery.

### 4.3. Rehabilitation of Muscle Synergy

Muscle synergy spatial patterns of the sampled post-stroke patients remained rather similar over subacute rehabilitation. Dominant muscles found with the highest relative activation in spatial patterns in each muscle synergy concurred with previous findings by Yang et al. (2017). In Synergy 1, corresponding to the initial lumbar flexion phase, the spatial activation of lumbar flexor RA dominated in upper body momentum generation. In Synergy 2, the ankle dorsiflexor TA was primarily activated to dorsiflex the ankle to the maximum position while moving the center of mass forward until the hip was raised from the seat. Synergy 3, responding for the body extension phase, was predominantly led by the knee extensor VAS and lumbar extensor ES. Both acted in extending the whole body during the upward momentum transition until the body reached the full upright position. For the last Synergy 4, the ankle planter-flexor SOL's spatial activation surpassed other muscles,' showing distinguished activation in stabilizing the body posture.

For the patient with the greatest motor recovery (FMA +13), both amplitude and timing of the patient's muscle synergy temporal patterns showed distinctive changes over the subacute rehabilitation. As shown in Figure 6, the patient's temporal pattern of normalized Synergy 2 exhibited a significant increase in peak amplitude on the second measurement day (95 days after stroke onset). After that, the amplitude escalation plateaued out. No apparent amplitude differences were found in the temporal pattern of normalized Synergy 2 between day 95, day 116, and day 144 after the stroke onset. Similarly, the peak time also seemed to advance toward an earlier time in the motion progress. This reduced lag in peak times, together with the increased peak amplitude in normalized Synergy 2 (for hip raise), may explain the patient's motor recovery since former studies have found that the peak time of Synergy 2 delayed significantly after stroke onset when compared to that of healthy controls (Yang et al., 2017, 2019). These former studies thereby insisted on teaching the patients the right time to raise their hips. According to the results, this study advocates such rehabilitation strategies targeted on unlearning incorrect ways of hip lifting. Additionally, the same patient (FMA +13) was the only one who showed significant amplitude increases in normalized Synergy 3. As in Figure 6, the peak level of temporal patterns in normalized Synergy 3 expanded more gradually, eventually reaching the highest peak amplitude by the last measurement day (144 days after stroke onset). It may suggest that apart from the timing features regarding Synergy 3 (Yang et al., 2019), growth in peak amplitude of Synergy 3 (body extension) may also be a distinctive feature indicating a better improvement in motor functioning.

For Synergy 4, besides the patient without any FMA improvement, the others exhibited significant peak amplitude increases. For instance, as in Figure 6, a continuously heightened peak amplitude in the temporal pattern of normalized Synergy 4 can be observed throughout the four measurements between day 25 and day 144 after stroke onset. A similar advancement of peak timing observed in Synergy 2 can also be seen in Synergy 4. The lessened delay in peak time and the increased peak amplitude in normalized Synergy 4 (for posture control) may be indicators of improvement in motor functioning, which may be used to discern motor recovery.

### 4.4. Limitations and Future Works

First, due to the generalized anthropometry in modeling different post-stroke participants, muscle tension estimated in the Hill-type muscle model may be sensitive to the change of some muscle geometry parameters. Both subject-specific musculoskeletal geometry and different neuromuscular control strategies have an impact on simulation results (Kainz et al., 2021). Although this study accounted for each participant's unique muscle activation when estimating muscle tension, it may be worth exploring further the impact of parameters, such as muscle fiber length and physiological cross-sectional area, on simulation results involving complex pathology (Redl et al., 2007). Second, the healthy and stroke groups are not age-matched in this study. Since anthropometry may not only change with pathology but also vary by age (Hicks et al., 2015), a future validation study with age-matched healthy participants may be necessary. Lastly, we followed the subacute rehabilitation of four inpatient stroke survivors on an average span of 4.5 months, and the number of patient participants is small. Current results may be indicative, but a larger pool of subacute patients is needed in order to draw stronger relevance between the observed biomechanical consequences and rehabilitation effectiveness (Yang et al., 2019). Recruiting more subacute stroke participants will also enable a future study to explain the current perplexing results found with less-improved patients, who may have experienced motor compensation instead of motor recovery.

## 5. Conclusion

This study proposed a novel method to compute post-stroke muscle activation based on joint torques. Upon validating the EMG-informed generic-scaled musculoskeletal model with eight healthy subjects, we applied it to investigate features related to muscle activation amplitude such as muscle tension and muscle synergy levels for four and three subacute stroke patients, respectively, during 137 ± 22 days of rehabilitation. In contrast to conventional EMG normalization methods, this joint torque-based normalization does not require MVC measurements or overestimate peak muscle activation in different muscles by indiscriminately scaling its peak to 100%, and hence activation amplitude comparisons can be made. Compared to the common SO algorithm based on squared muscle activation, our proposed algorithm based on joint torques produced results that were much closer to the MVC-normalized activation (virtual ground truth in this study). The contributed method and quantitative findings with patients of this study help enhance the understanding of post-stroke motor recovery mechanism and hyper-adaptability in humans with neurological disorders. It should also assist in the development of more effective rehabilitation strategies for future stroke survivors.

## Data Availability Statement

The original contributions presented in the study are included in the article/supplementary material, further inquiries can be directed to the corresponding author/s.

## Ethics Statement

The studies involving human participants were reviewed and approved by Institute Review Board of The University of Tokyo and the Institute Review Board of Morinomiya Hospital, Osaka, Japan. The patients/participants provided their written informed consent to participate in this study.

## Author Contributions

RW, QA, IM, and HA conceived the study. AY and HA supervised the research project. QA, HRY, AY, and HA developed the musculoskeletal model. QA, FA, and SS developed the measurement system. RW, QA, NY, HK, KY, HY, SS, HRY, MY, NH, KT, TF, and HO performed the measurement experiment and collected the data. NY and SS organized the database. RW, QA, and HH processed and analyzed the data. HH, SS, NH, IM, AY, and HA contributed critical review of the experiment procedure, physical therapy, patient rehabilitation, and method implementation. RW and QA wrote the manuscript. All authors read, revised, and approved the submitted manuscript.

## Funding

This research was supported by JSPS KAKENHI Grant Numbers 19H05729, 19K22799, and 18H01405.

## Conflict of Interest

The authors declare that the research was conducted in the absence of any commercial or financial relationships that could be construed as a potential conflict of interest.

## Publisher's Note

All claims expressed in this article are solely those of the authors and do not necessarily represent those of their affiliated organizations, or those of the publisher, the editors and the reviewers. Any product that may be evaluated in this article, or claim that may be made by its manufacturer, is not guaranteed or endorsed by the publisher.
